# Effects of Dietary Perilla Cake Supplementation in Growing Pig on Productive Performance, Meat Quality, and Fatty Acid Profiles

**DOI:** 10.3390/ani11113213

**Published:** 2021-11-10

**Authors:** Chaiwat Arjin, Chanmany Souphannavong, Rakkiat Norkeaw, Niraporn Chaiwang, Supamit Mekchay, Apinya Sartsook, Maninphan Thongkham, Thanchanok Yosen, Warintorn Ruksiriwanich, Sarana Rose Sommano, Korawan Sringarm

**Affiliations:** 1 Department of Animal and Aquatic Science, Faculty of Agriculture, Chiang Mai University, Chiang Mai 50200, Thailand; chaiwat_arjin@cmu.ac.th (C.A.); chanmany_so@cmu.ac.th (C.S.); rakkiat_nor@cmu.ac.th (R.N.); supamit.m@cmu.ac.th (S.M.); apinya_sat@cmu.ac.th (A.S.); Marninphan_t@cmu.ac.th (M.T.); 2 Department of Agricultural Technology and Development, Faculty of Agricultural Technology, Chiang Mai Rajabhat University, Chiang Mai 50300, Thailand; niraporn_cha@g.cmru.ac.th; 3 Cluster of Research and Development of Pharmaceutical and Natural Products Innovation for Human or Animal, Chiang Mai University, Chiang Mai 50200, Thailand; warintorn.ruksiri@cmu.ac.th (W.R.); sarana.s@cmu.ac.th (S.R.S.); 4 Central Laboratory, Faculty of Agriculture, Chiang Mai University, Chiang Mai 50200, Thailand; thanchanok.y@cmu.ac.th; 5 Department of Pharmaceutical Sciences, Faculty of Pharmacy, Chiang Mai University, Chiang Mai 50200, Thailand; 6 Cluster of Agro Bio-Circular-Green Industry (Agro BCG), Chiang Mai University, Chiang Mai 50200, Thailand; 7 Department of Plant and Soil Sciences, Faculty of Agriculture, Chiang Mai University, Chiang Mai 50200, Thailand

**Keywords:** fatty acids, grower pig, polyunsaturated fatty acid, omega-3, α-linolenic acid (ALA)

## Abstract

**Simple Summary:**

Perilla is an edible oil crop containing high levels of polyunsaturated fatty acids (PUFA), such as alpha-linolenic acid (ALA) and omega-3. The omega-3 helps in mitigating the risk of cardiovascular disease in humans. For industrial use, perilla seed is extracted for virgin oil, which is generally achieved by mechanical screw pressing. This process generates perilla cake that contains a fat content around 9–10%. In this study, we examined the effect of the supplementation of perilla cake in the pig diet on productive performance, pig carcass characteristics, meat quality, and fatty acid composition in fat tissue, and meat. The pig performance improved in terms of average daily gain after supplementation with perilla cake; however, the pig carcass and meat quality was unchanged. Moreover, the perilla cake supplement increased PUFA fatty acids and reduced the proportion of saturated fatty acids and unsaturated fatty acids in pork. The overall outcome of this study provides an alternative source of novel raw material for functional feed additives in livestock production.

**Abstract:**

The objective of this study was to determine the effect of perilla cake (PC) supplementation in a growing pig diet on overall growing performance, meat quality, and fatty acid profile. A total of 24 barrow grower crossbred pigs (Large White × Landrace) × Duroc with an initial average body weight of 26.33 kg were fed with a basal diet supplemented with PC at 0%, 5%, and 10% in (PC0, PC5, and PC10, respectively) for 12 weeks. At the end of the experimental period, pigs were slaughtered to determine carcass traits and meat quality. Back fat, abdominal fat, and *longissimus dorsi* (LD) muscle were collected to investigate fatty acid composition. The results show that the average daily gain (ADG) in the PC10 significantly increased. However, PC supplementation did not influence carcass traits and meat quality except the color as described by lightness (L*). Dietary PC supplementation significantly increased the α-linolenic acid (ALA, C18:3 cis-9, 12, 15), whereas n6/n3 ratio decreased significantly in all tissues investigated. Thus, it can be concluded that the supplementation of PC in growing pig diet is a potential way to increase the fatty acid composition to that required for healthier meat.

## 1. Introduction

Perilla (*Perilla frutescens* L.) is an annual herbaceous plant in the Lamiaceae family. It has been cultivated widely in China, India, Japan, Korea, Thailand, and in many other Asian countries as a source of edible oil, protein, and fiber [1] with various biological properties such as antiviral [2,3], anti-inflammatory [4], and antioxidant [2,5] effects. Perilla oil consists of 90.60% total unsaturated fatty acids, 17.90% monounsaturated fatty acid, and 72.70% polyunsaturated fatty acids [6]. The latter is in the form of omega-3 polyunsaturated fatty acids (PUFAs), specifically α-linolenic acids (ALA) 55.00–64.00% [7,8,9] and omega-6 and omega-9 fatty acids [7]. Presently, the production of perilla seed in Thailand accounts for roughly 272 tons/year of refined oil, and meal (ca. 60%) with low fat content, around 1–2% [10,11]. In addition, the screw press method is one of the popular techniques for extracting perilla oil, which yields perilla cake (PC) as a biomass. This cold pressing method, nonetheless, yields as much as ca. 8–14% of the available oil in the cake [12]. Souphannavong et al. [11] described that the PC contained crude protein (CP) 31.54%, ether extract (EE) 10.52%, and more importantly, high ALA ca. 55.97%. Thus, considering the functional ingredients present, it is interesting to use this biomass in animal diets to improve the overall quality of the livestock.

Pork is the most widely eaten meat in the world, but typical feeding practices lead to poor meat quality, as defined by a high omega-6 (n6) to omega-3 (n3) fatty acid ratio and low n3 fatty acid [13]. The conventional farmed pork also contains saturated fatty acids [14], which have adverse effects on human health. The ALA are essential for the normal growth and development of humans and animals [15,16], involved in the evolution of brain activity and the nervous system, and play an important role in the prevention and treatment of cardiovascular diseases, inflammatory diseases, and cancer [17,18]. Consumption of n3 PUFA from terrestrial animal products is mainly limited to the intake of ALA [19]. The main sources of long-chain n3 PUFA are marine fish, seafood, and fish oil [20]. Sadly, over 66.8% of the world’s adult population has very low intake of n3 PUFA as they are not able to access seafood [20]. One way to achieve the recommended daily intake of the n3 PUFA omega-3 fatty acids is to consume meat and meat products, and research is currently searching for the functional ingredients that can be supplemented in feed, thereby improving the n3 fatty acid composition in the meat [13,21,22]. For this reason, PC with high ALA content was a possible candidate for feed supplementation to improve the level of n3 PUFA in pigs. In previous work, Cui et al. [23] reported that chickens fed with perilla oil diets exhibited higher contents of α-linolenic acid (C18:3n3), DHA (22:6n3), polyunsaturated fatty acids, and n3 fatty acids, as well as a lower n6/n3 ratio. Oh et al. [24] also reported that feeding broilers with 2% perilla seed meal in their diet could improve growth performance, meat quality, and the fatty acid composition of thigh meat; specifically, the omega-3 fatty acid (7.55%) was higher than the control diet (6.64%). Hadi and Sudiyano [25] described that adding perilla seed meal to the diet of ducks increased average daily gain and omega-3 fatty acids (0.94%). Furthermore, Peiretti et al. [21] reported that perilla seeds supplementation in the rabbit diet increased α-linoleic acid and polyunsaturated fatty acid contents in the *longissimus dorsi* (LD) muscle. There is currently no study on the efficiency of supplementation of PC in pig diet in improving n3 PUFA. Consequently, the objective of this study is to investigate the effect of PC supplementation in the growing pig diet on the productive performance, meat quality, and fatty acids profile.

## 2. Materials and Methods

This study was carried out in strict accordance with the recommendations in the Guide for the Care and Use of Agricultural Animals in Research and Teaching. The experimental protocols were reviewed and approved by the Animal Care and Use Committee of Chiang Mai University (2560/AG-0001) prior to the experiment.

### 2.1. Animals and Management

This experiment was performed at the Mea Hia Agriculture Resource Demonstrative and Training Center, Faculty of Agriculture, Chiang Mai University, Chiang Mai, Thailand. A total of 24 barrows grower crossbred pigs (Large White × Landrace) × Duroc with an initial average body weight of 26.33 kg were used. The pigs were randomly allotted into three groups (*n* = 8 per group) and each individual pig was penned in an area of 2.0 m^2^ on the concrete floor, with drinking water and a feeding trough provided [26]. The granulated feed and water were given ad libitum. Prior to the experiment, all pigs were dewormed and vaccinated.

### 2.2. Diets

The three diets were based on maize, broken rice, rice bran, fish meal, and soybean meal, supplemented with a vitamin–mineral premix. The perilla cake was supplemented as 5% and 10% in feed formula. The diets included perilla cake supplementation in basal diet at 0% (PC 0 or control), 5% (PC 5), and 10% (PC 10) (Table 1), formulated to contain equal concentrations of metabolizable energy, crude protein (CP), minerals, and vitamins to meet the requirements for growing pigs according to the National Research Council (NRC) [27]. In addition, the measured fatty acid compositions of the three diet formulars are presented in Table 2.

### 2.3. Sample Collection

The body weight of the individual pigs was taken at the end of weeks 0, 4, 8, and 12. In addition, feed intake was recorded daily to calculate the productive performance, including average daily feed intake (ADFI), average daily gain (ADG), and feed conversion ratio (FCR). At the end of the experiment, all animals were off feed, with access to water, for 12 h prior to slaughter, and were transported to the Huay Kaew Slaughterhouse, Department of Livestock Development, Chiang Mai, Thailand within 20 min. After a minimum of 6 h resting time, pigs were euthanized via electrical stunning and exsanguination. All experimental procedures were carried out following the good manufacturing practices of the abattoir Thai Agricultural Standard TAS 9004-2004 [28]. Individual hot carcass weights were recorded. The *longissimus dorsi* muscle, abdominal fat, and back fat were collected from the right half-carcass, stored in a plastic bag at −20 °C for chemical analysis.

### 2.4. Assessment of Carcass Traits

After chilling at 4 °C for 24 h post-mortem, carcasses were weighed for chill carcass weight. Carcasses were dressed according to Thai and USDA styles into four lean cuts (picnic, boston, loin, and ham) [29]. The carcass dressing percentages were calculated by dividing the carcass weight by the live weight, obtained after fasting. The loin eye area was measured by tracking the surface area of the 10th rib of the LD muscle according to Santos et al. [30]. Point counting was over a 1 cm^2^ plastic grid (PCGP 1 cm^2^) made from a graph paper sheet with a transparent plastic sheet as a copy. The sum of the squares was performed to obtain the total area. In addition, the backfat thickness was measured at the 11th rib (including the skin) with a vernier caliper in cm of the LD muscle, according to Álvarez-Rodríguez and Teixeira [31].

### 2.5. Assessment of Meat Quality

The LD muscle was cut into 2.54 cm-thick slices [29]. Then, all samples were vacuum-packaged and stored at −20 °C until further analysis. The LD muscle underwent proximate analysis for percentages of moisture, ash, ether extract, and crude protein according to the association of official analytical collaboration (AOAC) [32]. The average pH value was determined in the LD muscle at 45 min and 24 h postmortem using a Testo 205—pH electrode/NTC temperature measuring instrument (Testo, Lenzkirch, Germany). Meat color was determined at 48 h postmortem on the surface area of the LD muscle after blooming for 1 h at 4 °C and fluorescent lighting at 2600 lumen [33]. The color values including L* (lightness), a* (redness), and b* (yellowness) were taken using a Minolta Chroma Meter Model CR-400 (Minolta Camera, Osaka, Japan). Then, the drip loss of the LD muscle was measured by preparing approximately 30 g of sample in a plastic bag and storing it at 4 °C for 24 h [34]. The water extruded from the sample was removed and the sample was weighed.

### 2.6. Fatty Acid (FA) Analysis

The diets, backfat, abdominal fat, and LD of growing pigs were analyzed and the fatty acid (FA) profile was analyzed according to the method of Chaiwang et al. [29]. Lipids were extracted from diets, backfat, abdominal fat, and LD by the Soxhlet extraction (method 920.39). Fatty acid methyl esters were prepared as described by Morrisson and Smith [35]. Gas chromatography–flame ionization detector (GC–FID) analysis was accomplished using the Shimadzu model GC-2030 (Kyoto, Japan) equipped with a 0.25 mm × 100 m × 0.25 µm wall-coated fused wax capillary column (RT-2560, RESTEK, Bellefonte, PA, USA). Helium was used as the carrier gas. Injector temperatures were held at 250 °C. Oven temperature programming was increased from 100 °C, held for 4 min, increased from 100 to 240 °C at a rate of 3 °C /min, and then held at 240 °C for 20 min. Injector volume was 1 µL, and the flame ionization detector temperature was 250 °C. Chromatograms were processed using the Lab Solution (Shimadzu, Kyoto, Japan). Identification was accomplished by comparing the retention time of peaks from samples with those of fatty acid methyl ester (FAME) standard mixtures (Food Industry Fame Mix, RESTEK, Bellefonte, PA, USA).

### 2.7. Statistical Analyses

The statistical analyses were performed using the SPSS software package (version 23.0 for Window, SPSS Inc., Chicago, IL, USA). Analysis of variance (ANOVA) was used to evaluate the effects of the PC supplementation in the diet on productive performance, carcass trait, meat quality, and fatty acid composition. Differences in means among treatment groups were determined using Duncan’s multiple range test, with a *p* < 0.05 indicating statistical significance.

## 3. Results

### 3.1. Chemical Composition and Fatty Acid Profile of PC

The chemical composition and fatty acid contents of PC are shown in Table 1 and Table 2. In the present study, PC was rich in crude protein (31.54%) and fat (10.52%), while it had a relatively high crude fiber content (24.43%). The addition of various levels of PC in the growing pig diet decreased linoleic acid (LA; C18:2 cis-9, 12) and significantly increased α-linolenic acid or ALA (C18:3 cis-9, 12, 15) content, while the n6/n3 ratio decreased with increasing PC inclusion levels (*p* < 0.05) (Table 2).

### 3.2. Productive Performance

The effect of PC supplementation in the growing pig diet on productive performance is presented in Table 3. The results show no significant difference among the treatments on the final weight, even though pigs in the PC10 group had a higher final weight than they did in other groups (*p* > 0.05). During weeks 0–4 of the experimental period, PC10 was the significantly highest FCR compared with PC0 and PC5 (2.81 vs. 2.56 and 2.50, respectively). In weeks 5–8 of the experiment, we found that ADFI of the PC10 group was significantly higher than the other groups during this period, and for the overall period (*p* < 0.05). In the last period of the experimental study (weeks 9–12), pigs fed with PC10 exhibited significantly higher ADG than the other groups (*p* < 0.05) However, in the overall experimental period, the PC10 group exhibited significantly higher ADFI and ADG (*p* < 0.05).

### 3.3. Carcass Traits and Meat Quality

The supplementation of PC in the growing pig diet did not affect pig carcass traits, including slaughter weight, carcass percentage, hot carcass weight, chill carcass weight, carcass length, and backfat thickness (*p* > 0.05) (Table 4). The meat quality, as described in terms of drip loss percentage, pH at 45 min and pH at 24 h, was not affected by the PC supplementation, except in terms of the meat color, especially the lightness (L*). The PC10 group expressed the lowest L* value (48.42) compared with the other groups. In addition, we found that the PC supplementation in the growing pig diet did not influence the chemical composition of LD muscle, including moisture, crude protein, ether extract, and ash (*p* > 0.05) (Table 5).

### 3.4. Fatty Acid Profiles in Backfat, Abdominal Fat, and Longisimus dorsi

To explore the effect of the supplementation of PC in the growing pig diet on the FA composition in pigs, we collected the tissues at three positions viz. back fat, abdominal fat, and LD muscle, and determined the FA profiles. We found that the supplementation of PC did not affect the fatty acids in the saturated fatty acid (SFA) and monounsaturated fatty acid (MUFA) compositions (*p* > 0.05) in the backfat (Table 6). However, the LA (C18:2 cis-9, 12) content in backfat significantly increased when the PC supplementation was at 10% (PC10). Moreover, PC supplementation correlated with a significantly higher ALA (C18:3 cis-9, 12, 15) compared with PC0 in backfat (*p* < 0.05). Similarly to the backfat, PC supplementation did not influence the SFA and MUFA compositions in the abdominal fat (Table 7). On the other hand, we found that the ΣSFA was significantly lower in the PC10 group than it was in the PC0 group. In addition, the ALA was increasingly raised with the PC supplementation levels (*p* < 0.05). For the LD fat, we found that the PC supplementation in the diet significantly decreased the SFA composition (*p* < 0.05) (Table 8), including palmitic acid (C16:0), stearic acid (C18:0), behenic acid (C22:0), and tricosylic acid (C23:0). Conversely, PC supplementation increased the unsaturated fatty acid composition significantly (*p* < 0.05), especially LA and ALA. Furthermore, the ΣSFA in LD fat was significantly lower in PC5 and PC10 compared with PC0. In addition, this study indicates that the supplementation of PC in the diet significantly increased ΣMUFA and ΣPUFA in LD fat (*p* < 0.05).

**Figure 1:**
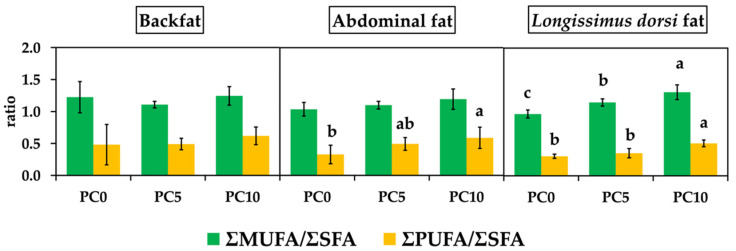
Effect of dietary perilla cake supplementation on the ratio of fatty acids in backfat, abdominal fat, and *longissimus dorsi* muscle. Superscription (**a**, **b** and **c**) indicates a significant difference between groups in ΣMUFA/ΣSFA and ΣPUFA/ΣSFA ratio (*p* < 0.05). PC0, control; PC5, 5% perilla cake supplementation; PC10, 10% perilla cake supplementation.

**Figure 2:**
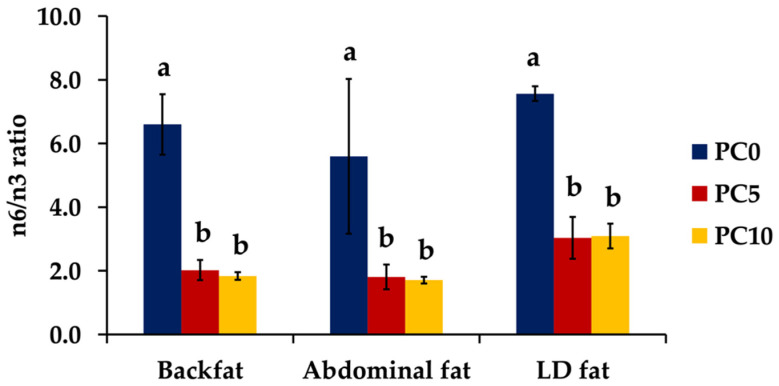
Effect of dietary perilla cake supplementation on the ratio of n6/n3 in backfat, abdominal fat, and *longissimus dorsi* muscle (LD). Superscription (**a** and **b**) indicates significant differences (*p* < 0.05) between groups in each fat tissue. PC0, control; PC5, 5% perilla cake supplementation; PC10, 10% perilla cake supplementation.

The ratios of ΣMUFA/ΣSFA and ΣPUFA/ΣSFA are illustrated in Figure 1. The supplementation of PC in the diet did not affect ΣMUFA/ΣSFA and ΣPUFA/ΣSFA in backfat. The ΣPUFA/ΣSFA ratio was significantly higher in the PC10 group in abdominal fat and LD fat (*p* < 0.05). Moreover, the n6/n3 ratio was lower (*p* < 0.05) in all tissues when supplementation was used in the diet (Figure 2).

## 4. Discussion

Generally, perilla is used for oil production as a rich source of omega-3 polyunsaturated fatty acids, specifically alpha-linolenic acid [7]. Perilla cake is a by-product of the perilla seed oil cold pressing process, and contains high protein content (31.54%), natural fatty acids, and dietary fiber [11]. In this study, we found that the EE content in PC was 10.52% which was in agreement with the findings of Shrikanta Rao [12], who reported that mechanical screw presses are relatively inefficient for edible oil recovery, leaving ca. 8–14% of the available oil in the cake. Additionally, the EE in the PC was higher than that of perilla meal, which was reported to contain ca. 1.08% of EE [38]. Souphannavong et al. [11] found that total PUFA was as high as 70.91%, which mostly consisted of the ALA (55.97%). Presently, the concern for food intake relating to health issues has led to the demand for functional foods such as diets rich in omega-3 fatty acids [39]. When pigs are fed diets with n3 fatty acids, pork and pork products could be recognized as a functional food with new health-promoting properties [40]. However, the use of PC in the diet should be considered at suitable supplement levels, because PC possesses relatively high fiber (24.43%) even if there is a high CP. Arjin et al. [26] explained that the high level of fiber in the diet leads to a low digestibility of nutrients. Therefore, the level of PC supplementation used in this experiment was in accordance with our previous study, in which the supplementation of PC in the growing pig diet was suggested to be no more than 10% [11]. In the present study, PC supplementation did not affect the final weight during the experimental period. However, in weeks 0–4 of the experimental period, the PC10 group exhibited significantly higher FCR levels than the other groups. There was a relationship between lower ADG and high ADFI in this period that led to the increase in FCR. Moreover, in the overall experimental period, the PC10 group was significantly higher in ADFI and ADG than in other groups (*p* < 0.05). Our results agree with several studies that reported that the supplementation of n3 PUFA (linseed) in the diet significantly increased indicators of productive performance, such as ADFI, ADG, and FCR [41,42,43].

The dietary treatments in this study did not affect the carcass traits, including slaughter weight, carcass percentage, hot carcass weight, chill carcass weight, carcass length, and backfat thickness (*p* > 0.05). These results agree with the reports of Tratarkoon et al. [44], Ivanovic et al. [45], and Bertol et al. [46], who reported that the supplementation of different fat sources did not affect carcass traits. In terms of the meat quality, PC supplementation in growing pig diets did not affect meat quality factors such as drip loss, pH at 45 min, pH at 24 h, and chemical composition. These finding were in line with de Tonnac and Mourot [47], who reported that the supplementation of n3 PUFA in the diet did not influence meat quality, especially pH and drip loss in the finishing pig. In addition, we noticed the higher range of pH at 24 h in LD. This indicates the dark firm dry (DFD) which occurs due to heat stress, as the experimental period was conducted during summer in Chiang Mai (March to June), Thailand with a high temperature range ca. 30-39°C. Regarding this, Adzitey and Nurul [37] explained that when animals were exposed to chronic or long-term stress before slaughtering, the DFD meat can occur. The examples of chronic stress are long distance transportation, long hours of food deprivation, and overcrowding of animals in the lairage over a long period of time [37]. However, the pH 45 min and pH 24 h were not significant different between the treatments. Nonetheless, we found that the color of meat, particularly lightness (L*) in PC10, was significantly lower than that in other groups. Tartrakoon et al. [44] explained that the increased high content of unsaturated fatty acids in the diet affects meat color, especially L*. The low L* values also indicate a low fat content [29]. Moreover, Filho et al. [48] showed that water holding capacity had a positive correlations with lightness (L*), despite the absence of correlations between L* and pH 45 min and the low negative correlations of L* with pH 24 h. Together with the pH fall, the denaturation of myofibrillar and sarcoplasmic (myoglobin) proteins and the expulsion of the water from the myofibrils towards the extracellular space during rigor mortis may lead to structural changes that increase light scattering, making the meat paler (greater L*) [48,49,50,51].

In the present study, the supplementation of PC in diet altered the fatty acid composition in pig tissues, including backfat, abdominal fat, and LD, mainly unsaturated fatty acids. Kouba and Mourot [52] reported that fatty acid composition in animal products is influenced both by the biosynthesis of fatty acids in animal tissues and by the lipids present in feedstuffs consumed by livestock. The effect of nutrition is more important in monogastric animals than in ruminants, because ruminants are capable of hydrogenating FA in the rumen. Moreover, Wojtasik et al. [19] explained that it was possible to change fatty acid content and the relationship between fatty acids that belonged to the n6 and n3 series in pig tissues, by applying an appropriate source of fat in the diet. In this study, the supplementation of PC in the diet significantly increased LA and ALA in all analyzed tissues. The results agree with the report of Nuernberg et al. [53], that the diet supplemented with 5% olive oil or 5% linseed oil significantly increased the relative content of linolenic acid and long-chain n3 fatty acids in lipids of muscle and backfat in pigs. The LA and ALA cannot be synthesized de novo because it lacks ω-6 (Δ12)-desaturase and ω-3 (Δ15)-desaturase, and, thus, they are essential dietary FA for humans and other mammals [20,54,55]. The PC was relatively rich in ALA (55.97%) content [11]. Therefore, we believe that this is one reason to induce high ALA content in tissues. However, the deposition of ALA depends on various factors, such as feed and type of tissue. Sobol et al. [56] found a higher ALA deposition in subcutaneous fat than in meat. They also reported that the deposition of ALA in pigs ranged from 50.4 to 69.0% in pigs weighing around 60–105 kg [56]. PC supplementation in the growing pig diet resulted in total ΣPUFA higher than in PC0 group for all analyzed tissues, but only LD was significantly different (*p* < 0.05), and similar results were presented by several studies; pig diet containing high n3 fatty acid contributes to a significantly higher ΣPUFA in various tissues [42,56,57,58]. We found that both LA and ALA were mainly fatty acids of ΣPUFA composition in the analyzed tissues. Generally, LA and ALA are metabolic precursors of long-chain n3 PUFA [20], including eicosapentaenoic acid (C20:5 or EPA) and docosahexaenoic acid (C22:6 or DHA). The biosynthetic pathway involves successive desaturation and chain elongation steps in the endoplasmic reticulum and a β-oxidation process localized in the peroxisome [20,59,60]. We found in this study that the supplementation of PC in the growing pig diet had no influence on DHA content in all analyzed tissues. This result agrees with Smink et al. [61], who reported that high ALA intake increased the long-chain ω-3 PUFA content, except that of DHA, in grower pigs. The increase in unsaturated fatty acids in the PC supplementation diet affected the ratio of ΣMUFA/ΣSFA significantly, being increased in abdominal fat, and increased the ΣPUFA/ΣSFA ratio in LD when dietary supplementation of PC was 10% in the diet. Our experimental results agree with Wojtasik et al. [19], in that the supplementation of n3 polyunsaturated fatty acids in diet influenced the increase in the ΣPUFA/ΣSFA ratio in LD muscle and subcutaneous fat. However, De Smet et al. [62] demonstrated that the ΣPUFA/ΣSFA ratio was influenced by genetics first, then followed by nutrition (mainly the overall fat level of the animal and intramuscular fat content). In addition, Cui et al. [23] reported that the supplementation of perilla seed oil induced a significantly reduced ΣPUFA/ΣSFA ratio in chicken breast muscle. In this study, the supplementation of PC 10% in the growing pig diet increased the ΣPUFA/ΣSFA ratio in backfat, abdominal fat, and LD to a higher level than that recommended by the UK Department of Health, and they recommended a ratio of ΣPUFA/ΣSFA in pork of 0.4 as a minimum [13]. Furthermore, the n6/n3 ratio was reduced in the investigated tissues when the level of PC supplementation was increased. The result showed the proportion of n6/n3 ratio between PC0 and PC supplementations (PC5 and PC10) ranged between 2.6 and 3.6 that was lower than the recommended ratio by the UK Department of Health (the n6/n3 recommended ratio is 4:1) [13]. Similar findings were obtained by Wojtasik et al. [19] and Okrouhlá et al. [63], who found that the increased n3 fatty acid content in the diet decreased the n6/n3 PUFA ratio. The decrease in the n6/n3 ratio means that the supplementation of PC in the diet increased the n3 fatty acid content in the pig. A previous explanation was that the fatty acid composition and ΣPUFA/ΣSFA and n6/n3 ratios in pig tissues are strongly influenced by the fat source in the diet [64]. This study found that a diet containing high ALA by PC supplementation had a significantly preferable influence on the fatty acid content in pig tissues, by increasing the level of ALA in tissues. The level of n3 fatty acid or ω-3 are important in mitigating the risk of cardiovascular diseases. Generally, the adult population has a very low intake of ω-3 PUFA due to limited access to the appropriate food sources, mainly seafood [20,65]. The improvement in pork quality by increasing n3 fatty acid content is necessary to help consumer meet the minimum nutritional requirement. Therefore, PC is a potential functional ingredient to enhance n3 fatty acid in pork. More importantly, the outcomes of this research encourages bio-circular green economic policy through biomass valorization.

## 5. Conclusions

Based on the results of this study, it can be concluded that the supplementation of perilla cake in the growing pig diet improved product performance, in particular, the average daily gain, without affecting carcass traits and meat quality, except the lightness. At the same time, the supplementation of this biomass in pig diet elevated the fatty acid compositions in backfat, abdominal fat, and the *longissimus dorsi* muscle. In addition, perilla cake supplementation enhanced the polyunsaturated fatty acid content, especially C18:3n3, in their tissues, as well as the ΣPUFA/ΣSFA and n6/n3 ratios. On the basis of the obtained results, perilla cake has the potential to be used in pig diet to enhance pork quality, as an alternative functional meat that has a high n3 fatty acid content for consumer health concerns. The mechanism for changing fatty acid deposition and its effects on the health status in pigs requires further investigation.

## Figures and Tables

**Table 1:** The diet formulation and nutritional values of perilla cake in growing pig diets.

Items	PC0	PC5	PC10
*Ingredient (%)*			
Maize	31.00	28.50	27.00
Broken rice	31.00	27.22	27.00
Rice barn	9.92	15.00	15.00
PC	0.00	5.17	10.34
Soybean meal 44 **%**	20.68	15.51	10.34
Fish meal 58 **%**	4.80	6.00	7.72
DCP	2.00	2.00	2.00
Salt	0.25	0.25	0.25
Premix ^a^	0.35	0.35	0.35
Total	100	100	100
*Chemical compositions*			
Dry matter, %	89.99	89.59	89.76
Crude protein, %	20.40	20.33	20.10
Ether extract, %	3.97	4.31	4.58
Ash, %	4.37	6.51	6.78
Crude fiber, %	22.94	25.49	28.43
Gross energy, Cal/g	4056	4400	4668
Digestible energy, Cal/g	4038	4021	3997
Metabolizable energy, Cal/g	3999	3958	3936
Net energy, Cal/g	3037	3001	2985
*Nutritive value (determined)*			
Lysine, %	1.12	1.13	1.12
Methionine, %	0.34	0.33	0.33
Threonine, %	0.72	0.72	0.73
Tryptophan, %	0.19	0.19	0.18

Mean of triplicate (*n* = 3) (chemical composition). PC0, control; PC5, 5% perilla cake supplementation; PC10, 10% perilla cake supplementation. ^a^ Vitamin premix (U or mg provided per kg of premix): vitamin A, 12,000 U; vitamin D3, 4500 U; vitamin E, 70 U; vitamin K, 3.5 mg; vitamin B1, 3 mg; vitamin B2, 7.5 mg; vitamin B3, 30 mg; vitamin B5, 65 mg; vitamin B6, 4.3 mg; vitamin B9, 2 mg; vitamin B12, 0.025 mg; biotin, 0.3 mg; choline chloride, 800 mg.

**Table 2:** Fatty acids composition of the experimental diets.

Fatty Acid (g/100g Fat)	Formula	PC0	PC5	PC10	SEM	*p*-Value
*Saturated fatty acid*						
Miristic acid	C14:0	0.32 ^b^	0.56 ^a^	0.58 ^a^	0.041	0.002
Pentadecylic acid	C15:0	0.08 ^b^	0.13 ^a^	0.14 ^a^	0.010	0.034
Palmitic acid	C16:0	15.55	19.78	18.27	0.886	0.139
Heptadecanoic acid	C17:0	0.18 ^b^	0.26 ^a^	0.26 ^a^	0.015	0.044
Stearic acid	C18:0	3.30 ^b^	5.08 ^a^	4.90 ^a^	0.284	0.003
Arachidic acid	C20:0	0.87	0.90	0.85	0.040	0.888
Heneicosylic acid	C21:0	0.01	0.02	0.01	0.003	0.606
Behenic acid	C22:0	0.62 ^a^	0.50 ^ab^	0.43 ^b^	0.033	0.026
Tricosylic acid	C23:0	0.24	0.22	0.28	0.014	0.333
Lignoceric acid	C24:0	1.04	0.81	0.77	0.183	0.070
*Monounsaturated fatty acid*						
Pentadecanoic acid	C15:1	0.03	0.04	0.04	0.002	0.202
Palmitoleic acid	C16:1 (cis-9)	0.54 ^b^	0.76 ^a^	0.79 ^a^	0.043	0.015
Heptadecanoic acid	C17:1	0.05	0.06	0.06	0.003	0.653
Oleic acid	C18:1 (cis-9)	40.58	34.87	31.35	1.968	0.155
Gondoic acid	C20:1	0.42	0.38	0.37	0.019	0.571
Erucic acid	C22:1	0.03	0.02	0.03	0.007	0.520
Nervonic acid	C24:1	0.11	0.15	0.14	0.009	0.123
*Polyunsaturated acid*						
Linoleic acid	C18:2 (cis-9,12)	32.39	27.49	27.69	1.601	0.406
Eicosadienoic acid	C20:2 (cis-11,14)	0.40	0.56	0.44	0.072	0.671
Docosadienoic acid	C22:2	0.03	0.03	0.03	0.002	0.856
γ-linolenic acid	C18:3 (cis-6,9,12)	0.03 ^c^	0.05 ^b^	0.08 ^a^	0.007	0.000
α-linolenic acid	C18:3 (cis-9,12,15)	1.27 ^c^	5.73 ^b^	10.61 ^a^	1.191	0.000
Dihomo-γ-linolenic acid	C20:3	0.03	0.02	0.02	0.003	0.438
Docosapentaenoic acid	C20:5	0.34	0.27	0.35	0.019	0.136
Docosahexaenoic acid	C22:6	1.20	0.92	1.20	0.701	0.167
ΣSFA		22.26	28.33	26.54	1.268	0.132
ΣMUFA		41.74	36.26	32.78	1.996	0.187
ΣPUFA		35.30	34.53	40.02	1.957	0.507
ΣMUFA/ΣSFA		1.87 ^a^	1.27 ^b^	1.23 ^b^	0.088	0.000
ΣPUFA/ΣSFA		1.58 ^a^	1.20 ^b^	1.51 ^a^	0.053	0.000
C18:2 n6/C18:3 n3		25.56 ^a^	4.81 ^b^	2.61 ^c^	3.118	0.000
n6/n3		11.58 ^a^	4.01 ^b^	2.29 ^c^	1.219	0.000

Different superscripts (^a, b and c^) in the same row indicate a significant difference (*p* < 0.05). PC0, control; PC5, 5% perilla cake supplementation; PC10, 10% perilla cake supplementation; SEM, standard error of mean; SFA, saturated fatty acid; MUFA, monounsaturated fatty acid; PUFA, polyunsaturated.

**Table 3:** Effect of dietary perilla cake supplementation on the productive performance of growing pigs.

Items	PC0	PC5	PC10	SEM	*p*-Value
Initial weight, kg	25.87	27.14	25.98	0.622	0.673
Final weight, kg	69.00	67.00	73.00	1.492	0.198
*Weeks 0*–*4 of the experimental periods*					
ADFI, kg/day	1.26	1.36	1.35	0.026	0.245
ADG, kg/day	0.51	0.56	0.48	0.017	0.159
FCR	2.56 ^ab^	2.50 ^b^	2.81 ^a^	0.052	0.024
*Weeks 5*–*8 of the experimental periods*					
ADFI, kg/day	1.42 ^ab^	1.33 ^b^	1.57 ^a^	0.039	0.032
ADG, kg/day	0.52	0.53	0.60	0.022	0.249
FCR	2.81	2.67	2.76	0.038	0.297
*Weeks 9*–*12 of the experimental periods*					
ADFI, kg/day	1.64	1.62	1.60	0.107	0.986
ADG, kg/day	0.34 ^b^	0.38 ^ab^	0.50 ^a^	0.027	0.017
FCR	4.48	4.47	4.11	0.425	0.932
*All of the experimental period*					
ADFI, kg/day	1.50 ^ab^	1.43 ^b^	1.64 ^a^	0.036	0.028
ADG, kg/day	0.48 ^b^	0.48 ^b^	0.55 ^a^	0.012	0.019
FCR	3.36	3.25	3.24	0.168	0.959

Different superscripts (^a and b^) in the same row indicate a significant difference (*p* < 0.05). PC0, control; PC5, 5% perilla cake supplementation; PC10, 10% perilla cake supplementation; SEM, standard error of mean.

**Table 4:** Effect of dietary perilla cake supplementation on the carcass traits and meat quality of growing pigs.

Items	PC0	PC5	PC10	SEM	*p*-Value
*Carcass traits*					
Slaughter weight, kg	69.00	67.00	73.00	1.492	0.198
Carcass percentage, %	65.40	69.12	65.84	2.889	0.903
Hot carcass, kg	48.63	49.85	50.50	1.429	0.913
Chilled carcass weight, kg	45.24	46.16	48.30	2.486	0.921
Carcass length, cm	76.00	78.50	84.00	1.979	0.282
Back fat thickness, cm	1.11	1.12	1.16	0.061	0.955
*Meat quality*					
Drip loss, **%**	7.08	5.76	5.26	0.391	0.104
pH 45 min	6.33	6.32	6.55	0.063	0.228
pH 24 h	6.00	6.06	6.00	0.027	0.652
color					
L*	52.76 ^a^	50.21 ^ab^	48.42 ^b^	0.849	0.041
a*	5.48	5.06	5.05	0.446	0.939
b*	5.85	6.87	6.56	0.364	0.617

Different superscripts (^a and b^) in the same row indicate a significant difference (*p* < 0.05). PC0, control; PC5, 5% perilla cake supplementation; PC10, 10% perilla cake supplementation; SEM, standard error of mean; The pH 24 h of a normal muscle ranges from 5.50 to 5.80, and dark firm dry (DFD) meat is > 6.00 [36,37]; L*, lightness; a*, redness; b*, yellowness.

**Table 5:** Effect of dietary perilla cake supplementation on the chemical composition of the *Longissimus dorsi* of growing pigs.

Chemical Composition (%)	PC0	PC5	PC10	SEM	*p*-Value
Moisture	71.38	73.33	73.58	0.609	0.297
Crude protein	22.72	22.33	22.39	0.157	0.604
Ether extract	2.95	2.90	2.93	0.063	0.965
Ash	1.12	1.14	1.16	0.101	0.472

PC0, control; PC5, 5% perilla cake supplementation; PC10, 10% perilla cake supplementation; SEM, standard error of mean.

**Table 6:** Effect of dietary perilla cake supplementation on the fatty acid profile in the backfat of growing pigs.

Fatty Acid (g/100g Fat)	Formula	PC0	PC5	PC10	SEM	*p*-Value
*Saturated fatty acid*						
Miristic acid	C14:0	1.74	1.72	1.59	0.038	0.245
Palmitic acid	C16:0	18.47	18.17	17.28	0.339	0.350
Heptadecanoic acid	C17:0	1.89	nd	0.15	0.424	0.130
Stearic acid	C18:0	14.63	16.50	13.90	0.481	0.069
Arachidic acid	C20:0	0.23	0.17	0.19	0.026	0.637
Heneicosylic acid	C21:0	0.04	0.05	0.07	0.009	0.338
Behenic acid	C22:0	0.07	0.09	0.10	0.012	0.637
Tricosylic acid	C23:0	1.42	1.81	1.89	0.132	0.305
*Monounsaturated fatty acid*						
Palmitoleic acid	C16:1 (cis-9)	5.31	4.33	4.99	0.180	0.068
Heptadecanoic acid	C17:1	1.07	nd	0.05	0.311	0.302
Oleic acid	C18:1 (cis-9)	37.01	37.67	37.28	0.510	0.882
Gondoic acid	C20:1	1.36	0.65	1.04	0.177	0.276
*Polyunsaturated acid*						
Linoleic acid	C18:2 (cis-9,12)	9.27 ^b^	11.34 ^ab^	15.25 ^a^	1.001	0.037
Eicosadienoic acid	C20:2 (cis11,14)	4.44	0.72	1.09	0.987	0.247
γ-linolenic acid	C18:3 (cis-6,9,12)	0.03	0.02	0.02	0.012	0.863
α-linolenic acid	C18:3 (cis-9,12,15)	0.68 ^b^	3.70 ^a^	3.55 ^a^	0.049	0.005
Eicosatrienoic acid	C20:3 (cis-11, 14, 17)	1.99	2.17	1.97	0.148	0.840
Eicosapentaenoic acid	C20:5	0.26	0.08	0.06	0.046	0.166
Docosahexaenoic acid	C22:6	0.07	0.08	0.09	0.009	0.735
ΣSFA		38.51	38.50	35.14	1.041	0.330
ΣMUFA		44.75	44.44	43.86	0.640	0.859
ΣPUFA		16.74	17.06	21.00	1.197	0.284

Different superscripts (^a and b^) in the same row indicate a significant difference (*p* < 0.05). PC0, control; PC5, 5% perilla cake supplementation; PC10, 10% perilla cake supplementation; SEM, standard error of mean; nd, not detected lower than the limit of detection (0.01µg/mL); SFA, saturated fatty acid; MUFA, monounsaturated fatty acid; PUFA, polyunsaturated fatty acid.

**Table 7:** Effect of dietary perilla cake supplementation on the fatty acid profile in the abdominal fat of growing pigs.

Fatty Acid (g/100g Fat)	Formula	PC0	PC5	PC10	SEM	*p*-Value
*Saturated fatty acid*						
Miristic acid	C14:0	1.58	1.78	1.64	0.039	0.103
Palmitic acid	C16:0	17.12	18.53	14.47	0.351	0.248
Heptadecanoic acid	C17:0	0.36	1.27	0.15	0.425	0.546
Stearic acid	C18:0	14.13	16.44	14.77	0.462	0.104
Arachidic acid	C20:0	0.22	0.21	0.10	0.027	0.124
Heneicosylic acid	C21:0	0.06	0.04	0.06	0.008	0.411
Behenic acid	C22:0	0.16	0.21	0.51	0.077	0.130
Tricosylic acid	C23:0	1.52	1.81	1.62	0.117	0.621
*Monounsaturated fatty acid*						
Palmitoleic acid	C16:1 (cis-9)	4.76	4.43	4.69	0.193	0.788
Heptadecanoic acid	C17:1	0.99	nd	0.05	0.312	0.362
Oleic acid	C18:1 (cis-9)	36.42	36.64	37.07	0.487	0.870
Gondoic acid	C20:1	1.46	0.66	1.03	0.181	0.197
*Polyunsaturated fatty acid*						
Linoleic acid	C18:2 (cis-9,12)	10.57	10.04	13.19	0.954	0.344
Eicosadienoic acid	C20:2 (cis-11,14)	4.19	0.84	0.82	0.997	0.272
γ-linolenic acid	C18:3 (cis-6,9,12)	0.03	0.02	0.02	0.012	0.880
α-linolenic acid	C18:3 (cis-9,12,15)	1.49 ^b^	3.74 ^a^	3.90^a^	0.458	0.046
Eicosatrienoic acid	C20:3	2.17	2.33	2.38	0.133	0.825
Eicosapentaenoic acid	C20:5	0.61	0.73	0.35	0.072	0.078
Docosahexaenoic acid	C22:6	0.20	0.25	0.15	0.062	0.797
ΣSFA		42.42 ^a^	38.58 ^ab^	36.34 ^b^	1.058	0.034
ΣMUFA		43.64	41.74	42.85	0.689	0.549
ΣPUFA		13.93	19.69	20.81	1.292	0.059

Different superscripts (^a and b^) in the same row indicate a significant difference (*p* < 0.05). PC0, control; PC5, 5% perilla cake supplementation; PC10, 10% perilla cake supplementation; SEM, standard error of mean; nd, not detected lower than the limit of detection (0.01 µg/mL); SFA, saturated fatty acid; MUFA, monounsaturated fatty acid; PUFA, polyunsaturated fatty acid.

**Table 8:** Effect of dietary perilla cake supplementation on the fatty acid profile in the *Longissimus dorsi* muscle of growing pigs.

Fatty Acid (g/100g Fat)	Formula	PC0	PC5	PC10	SEM	*p*-Value
*Saturated fatty acid*						
Miristic acid	C14:0	1.11	1.24	1.01	0.046	0.114
Palmitic acid	C16:0	21.14 ^a^	19.14 ^b^	17.00 ^c^	0.527	0.001
Heptadecanoic acid	C17:0	1.10	1.04	0.96	0.047	0.494
Stearic acid	C18:0	17.07 ^a^	15.97 ^b^	14.05 ^b^	0.390	0.002
Arachidic acid	C20:0	0.40	0.46	0.41	0.085	0.959
Behenic acid	C22:0	0.21 ^a^	0.13 ^b^	0.08 ^b^	0.016	0.000
Tricosylic acid	C23:0	2.96 ^a^	1.96 ^b^	1.92 ^b^	0.160	0.004
*Monounsaturated fatty acid*						
Palmitoleic acid	C16:1 (cis-9)	4.32 ^b^	6.45 ^a^	6.30 ^a^	0.286	0.001
Heptadecanoic acid	C17:1	0.58 ^a^	0.39 ^b^	0.49 ^ab^	0.031	0.038
Oleic acid	C18:1 (cis-9)	36.29	37.89	38.94	0.457	0.056
Gondoic acid	C20:1	1.24	1.10	1.50	0.073	0.078
*Polyunsaturated fatty acid*						
Linoleic acid	C18:2 (cis-9,12)	10.48 ^b^	13.12 ^a^	12.93^a^	0.458	0.018
Eicosadienoic acid	C20:2 (cis-11,14)	0.76	0.87	1.03	0.049	0.073
γ-linolenic acid	C18:3 (cis-6,9,12)	0.11 ^ab^	0.14 ^a^	0.06^b^	0.013	0.035
α-linolenic acid	C18:3 (cis-9,12,15)	1.48 ^b^	3.29 ^a^	3.42 ^a^	0.251	0.000
Dihomo-γ-linolenic acid	C20:3	0.42 ^a^	0.33 ^b^	0.30 ^b^	0.017	0.004
Eicosapentaenoic acid	C20:5	0.11	0.13	0.14	0.010	0.602
Docosahexaenoic acid	C22:6	0.35	0.31	0.44	0.037	0.356
ΣSFA		44.01 ^a^	39.97 ^b^	35.43 ^c^	0.891	0.000
ΣMUFA		42.49 ^b^	45.84 ^a^	46.56 ^a^	0.606	0.005
ΣPUFA		13.50 ^b^	14.23 ^b^	18.03 ^a^	0.659	0.004

Different superscripts (^a, b and c^) in the same row indicate a significant difference (*p* < 0.05). PC0, control; PC5, 5% perilla cake supplementation; PC10, 10% perilla cake supplementation; SEM, standard error of mean; SFA, saturated fatty acid; MUFA, monounsaturated fatty acid; PUFA, polyunsaturated fatty acid.

## Data Availability

The data supporting the reported results are in the possession of the authors (C.A. and K.S.).
